# Epigenetic Regulation of Floral Transition

**DOI:** 10.3390/plants14223471

**Published:** 2025-11-14

**Authors:** Yulong Li, Dian Zhang, Jin Wang, Meiru Yang, Zhancai Yin, Keming Zhu, Yuanxue Liang, Xiaoli Tan

**Affiliations:** School of Life Sciences, Jiangsu University, Zhenjiang 212013, China; liyulongcas@163.com (Y.L.); zhangdian9988@163.com (D.Z.); jinwang@ujs.edu.cn (J.W.); 19931325584@163.com (M.Y.); y1514819820@163.com (Z.Y.); uegzkg@sina.com (K.Z.)

**Keywords:** flowering, epigenetics, chromatin remodeling, m^6^A modification, floral transition

## Abstract

As an emerging field of life science, epigenetics plays a pivotal role in regulating gene expression. Epigenetic modifications including histone modifications, DNA methylation, chromatin remodeling, non-coding RNAs, and RNA modifications (particularly m^6^A methylation) play crucial roles in fine-tuning plant developmental processes. Among these, floral transition marks a key developmental switch from vegetative to reproductive growth, orchestrated by complex interactions between endogenous signals (such as age and hormones) and environmental cues (such as photoperiod and temperature). Recent advances have uncovered that epigenetic mechanisms act as molecular bridges integrating these signals to ensure flowering occurs under optimal conditions. This review synthesizes the current understanding of epigenetic control in the six canonical flowering pathways—photoperiod, vernalization, autonomous, thermosensory, gibberellin, and age-dependent pathways—with a particular emphasis on the emerging role of m^6^A RNA modification. We also discuss the crosstalk among epigenetic layers and highlight the translational potential of epigenetic engineering in optimizing flowering time and crop adaptation.

## 1. Introduction

The precise regulation of flowering time is essential for plant reproductive success and agricultural productivity. In plants, gene expression is governed by the interplay among DNA, RNA, and proteins, which operate through multilayered regulatory hierarchies [1]. DNA carries genetic information and exists in eukaryotes as nucleosomes, which wrap around histone octamers [2]. Nucleosomes form bead-like structures that fold to create chromatin [3]. DNA and histone proteins undergo diverse covalent modifications, such as methylation, acetylation, and ubiquitination, that critically influence chromatin architecture and transcriptional accessibility [4]. RNA, as a bridge for gene expression, undergoes extensive post-transcriptional modifications to achieve functional maturity. Approximately two-thirds of all RNA chemical modifications are methylation [5]. Crucially, the combinatorial effects of these epigenetic modifications and three-dimensional chromatin organization collectively orchestrate spatiotemporal gene regulation [6]. This complex interplay of regulatory mechanisms, termed epigenetic regulation, enables heritable phenotypic variations independent of DNA sequence alterations—a phenomenon first conceptualized by Waddington in 1939 as “epigenetics” [7,8]. Contemporary research has identified five principal epigenetic mechanisms: DNA methylation, histone post-translational modifications, chromatin remodeling, non-coding RNA-mediated regulation, and RNA epitranscriptomics [9]. Collectively, these processes orchestrate developmental transitions and environmental responses by modifying chromatin accessibility and transcript stability [10,11]. Flowering represents a critical phenological transition for plants, indicating the irreversible shift from vegetative growth to reproductive development [12]. The process of flowering can be divided into three distinct stages: the floral induction, the floral primordia formation, and the floral organ development [13,14]. The floral induction serves as the developmental checkpoint integrating endogenous signals (e.g., hormonal status, age) and environmental cues (e.g., photoperiod, temperature) to ensure reproductive success. Current models propose six evolutionarily conserved flowering regulation pathways in plants: photoperiod-dependent pathway, vernalization pathway, autonomous pathway, thermosensory pathway, gibberellic-mediated pathway, and age-dependent pathway [13,15,16,17,18,19]. These pathways converge on fundamental group floral integration genes, including *FLOWERING LOCUS T* (*FT*) and *SUPPRESSOR OF OVEREXPRESSION OF CONSTANS 1* (*SOC1*), which activate downstream floral meristem identity genes such as *APETALA1* (*AP1*), *APETALA2* (*AP2*), *FRUITFUL* (*FUL*), and *LEAFY* (*LFY*) genes to ultimately trigger the flowering process in plants [20,21]. The precise temporal control of flowering has profound agronomic implications, directly influencing crop yield stability, harvest quality, and adaptation to changing climatic conditions. For instance, optimizing flowering synchronization can significantly improve resource allocation efficiency in cereal crops while enabling effective implementation of crop rotation systems (e.g., rice-oilseed rotations) through temporal niche partitioning [22,23]. Emerging evidence highlight that epigenetic regulation fine-tunes this transition by coordinating environmental perception with developmental programs. This review aims to emphasize the significance of epigenetic regulation in flowering induction and explore its potential applications in future agricultural production and crop breeding.

## 2. Overview of Flowering Regulation in Plants

The regulatory networks described are primarily derived from the model plant *Arabidopsis thaliana*, which serves as a foundation for understanding conserved and divergent mechanisms across species. While the genetic framework of flowering has been extensively characterized in Arabidopsis, homologous components with species-specific adaptations exist in monocots and perennials, such as the VRN1–VRN2–VRN3 module in cereals or Hd3a/RFT1 in rice. Each flowering pathway integrates unique environmental and developmental inputs, yet all ultimately converge at FT and SOC1. The following sections outline the major genetic circuits underlying these pathways, setting the stage for the subsequent discussion on their epigenetic regulation.

### 2.1. Photoperiod Pathway

The photoperiod pathway constitutes an evolutionarily conserved mechanism enabling plants to coordinate developmental transitions with periodic light–dark cycles. Central to this process is the endogenous circadian oscillator—a self-sustained 24 h molecular timekeeping system that synchronizes metabolic and physiological processes (e.g., flowering induction, stomatal dynamics, and nyctinastic movements) with external photoperiodic cues [24]. The photoperiodic flowering cascade comprises three functionally integrated modules: (1) light signal perception through photoreceptor complexes, (2) circadian clock-mediated signal processing, and (3) developmental output generation via florigenic signaling [25].

In angiosperms, leaf-localized photoreceptors, such as phytochromes (PHYA-E) absorbing red/far-red light and cryptochromes (CRY1/2) detecting blue/UV-A wavelengths, initiate signal transduction by perceiving photoperiodic variations [26,27]. These optical inputs are transmitted to the central circadian oscillator, a transcriptional-translational feedback loop involving core regulators CIRCADIAN CLOCK ASSOCIATED 1 (CCA1), LATE ELONGATED HYPOCOTYL (LHY), and TIMING OF CAB EXPRESSION 1 (TOC1) [28,29,30]. The entrained oscillator generates rhythmic outputs that gate light-responsive pathways, culminating in photoperiod-dependent activation of florigen synthesis.

A pivotal regulatory node is found in the diel oscillation of *CONSTANS* (*CO*) expression. The circadian-regulated nuclear protein GIGANTEA (GI) stabilizes *CO* transcripts through direct RNA-binding activity, establishing a dawn-specific expression peak under inductive long-day conditions [31]. CO, a B-box zinc finger transcription factor, induces the transcription of systemic florigen FLOWERING LOCUS T (FT) in phloem companion cells [32]. FT protein subsequently undergoes symplastic trafficking to the shoot apical meristem (SAM), where it interacts with the bZIP transcription factor FD to form a florigen activation complex (FAC) [33]. This complex transcriptionally activates meristem identity genes such as *APETALA1* (*AP1*) and *LEAFY* (*LFY*), thereby reprogramming SAM fate from vegetative to reproductive development [33,34].

Notably, in the GI-CO-FT module, PHYB-mediated phosphorylation targets CO for 26S proteasome degradation in darkness, ensuring light-specific accumulation [34]. FT mobility establishes a long-range signaling gradient, synchronizing flowering initiation across plant tissues [35]. This multilayered regulation ensures precise spatiotemporal control of flowering, exemplifying how photoperiodic inputs are transduced into developmental decisions through integrated molecular networks.

### 2.2. Vernalization Pathway

Vernalization is an epigenetic process whereby plants require extended exposure to low temperature before transitioning from vegetative to reproductive growth. This adaptation ensures flowering occurs at the appropriate period, aligning with favorable seasonal conditions [36]. The vernalization pathway is primarily regulated by two key genes: *FLOWERING LOCUS C* (*FLC*) and *FRIGIDA* (*FRI*) [37]. FLC, a MADS-box transcription factor, acts as a master floral repressor by directly binding to promoters of key flowering integrators such as SUPPRESSOR OF OVEREXPRESSION OF CONSTANS 1 (SOC1) and FLOWERING LOCUS T (FT), as well as the first intron of FD, thereby blocking their transcription. FRI, a plant-specific protein, serves as a scaffold protein that forms a transcriptional activation complex with FRI-IKE 1 (FRL1), FRI ESSENTIAL 1 (FES1), FRI SUPPRESSOR of FRI 4 (SUF4), and FLC EXPRESSOR (FLX) to activate the expression of the flowering repression gene *FLC* [38].

### 2.3. Autonomous Pathway

The autonomous pathway enables plants to flower independently of environmental cues, relying instead on internal developmental signals. This pathway primarily functions by suppressing *FLC* expression via RNA processing and chromatin remodeling mechanisms, ensuring flowering occurs at an appropriate developmental stage. Currently, the key genes identified in the autonomous pathway mainly encode two types of proteins: RNA-binding proteins such as FLOWERING LOCUS CA (FCA), FLOWERING LOCUS Y (FY), FLOWERING LOCUS KH DOMAIN (FLK), FLOWERING LOCUS PA (FPA), and TATA-binding protein-associated factor 15b (TAF15b); and chromatin remodeling factors like FLOWERING LOCUS VE (FVE), LUMINIDEPENDENS (LD), FLOWERING LOCUS D (FLD), and RELATIVE OF EARLY FLOWERING 6 (REF6) [39,40,41,42,43,44,45,46]. FCA and FPA encode RNA recognition motif (RRM)-containing proteins that process FLC precursor mRNA, reducing its stability and transcriptional output. FY interacts with FCA to regulate the 3′ end processing of FLC mRNA, further enhancing its degradation. FLK, containing a KH RNA-binding domain, post-transcriptionally silences *FLC* through mRNA processing. Unlike other flowering pathways, the autonomous pathway operates in a non-redundant manner, with its components functioning independently rather than forming a cascade amplification network.

### 2.4. Thermosensory Pathway

Ambient temperature affects flowering time through the thermosensory pathway, which integrates temperature signals with developmental cues. The core component of this pathway is FLOWERING LOCUS T (FT), a central floral integrator, whose expression is dynamically regulated by temperature fluctuations. In *Arabidopsis thaliana*, warming from 23 °C to 27 °C can promote the expression of *FT* and induce flowering [47]. The high-temperature accumulated transcription factor PHYTOCHROME INTERACTING FACTOR 4(PIF4) activates FT transcription by binding to FT promoters, facilitating flowering [48]. Additionally, FT is influenced by the MADS-box proteins FLOWERING LOCUS M (FLM) and SHORT VEGETATIVE PHASE (SVP). Temperature-sensitive splicing of FLOWERING LOCUS M (FLM) generates two isoforms, FLM-β and FLM-δ, with FLM-β being the predominant form at 16 °C. FLM and SVP form a complex that represses *FT* transcription by interacting with *FT* promoter motifs. When the ambient temperature increases from 16 °C to 27 °C, FLM-δ becomes the main form. The SVP-FLM-δ complex hinders the transcriptional inhibition of *FT* by SVP, thereby accelerating plant flowering [49]. Research indicates that microRNAs (miRNAs) are also involved in temperature-mediated regulation of the flowering process, including *miR156*, *miR172*, *miR163*, *miR169*, and *miR399* [50]. Among these, *miR156* plays a major role. With rising temperatures, the expression of *miR156* diminishes, leading to an increase in the expression of its target *SQUAMOSA PROMOTER BINDING-LIKE* (*SPL*) gene. The miR156-SPL complex can regulate FT by sensing temperature changes, thereby affecting flowering [51].

### 2.5. Gibberellin Pathway

Gibberellins (GAs) are diterpenoid plant hormones that are crucial for the induction of flowering, particularly in non-inductive short-day situations. [52]. The GA-mediated pathway promotes flowering through two main mechanisms: (1) Direct activation of floral integrators: Active exogenous GAs can up-regulate the expression of *LFY* or *SOC1* under short-day circumstances, and facilitating the production of AP1, thus promoting flowering [53,54]. GA can also promote flowering under long-day (LD) conditions by enhancing the expression of *FT* [55]. (2) DELLA protein degradation: The DELLA proteins is an important transcriptional repressor that inhibits flowering in the gibberellin pathway [56]. In *Arabidopsis thaliana*, the DELLA proteins mainly consist of GA INSENSITIVE (GAI), REPRESSOR OF GAL-3 (RGA), RGA-LIKE 1 (RGL1), RGA-LIKE 2 (RGL2), and RGA-LIKE 3 (RGL3). The gibberellin signal sensed by GA-INSENSITIVE DWARF1 (GID1) in plant leaves triggers ubiquitin-mediated degradation of the DELLA proteins, releasing PIF4 and SPL transcription factors to activate FT and LFY, thereby regulating plant flowering [57]. DELLA proteins can also interact with CO, a core protein in the photoperiodic pathway, integrating GA signaling with light-dependent flowering regulation to affect the flowering time [58].

### 2.6. Age Pathway

The age pathway ensures that plants transition to flowering only after reaching a specific developmental stage, integrating endogenous cues with environmental signals. This pathway is primarily regulated by the miR156-SPL-miR172 module: (1) miR156-SPL Axis. *miR156* concentrations are elevated in immature plants, maintaining vegetative growth by repressing SQUAMOSA PROMOTER BINDING-LIKE (SPL) transcription factors. As plants mature, *miR156* levels decline, enabling SPL proteins to activate *miR172* expression. In *Arabidopsis thaliana*, *miR156* targets SPL2, SPL9, SPL10, SPL11, SPL13, SPL15, which are associated with flowering, and SPL3, SPL4, SPL5, which promote flower meristem differentiation [59]. Among them, SPL9 and SPL15 promote *miR172* expression by interacting with its promoter. (2) miR172-AP2 (APETALA2) Axis. *miR172* targets AP2-like transcription factor members TARGET OF EARLY ACTIVATION TAGGED 1 (TOE1), TOE2, SCHNARCHZAPFEN (SNZ), and SCHLAFMUTZE (SMZ), relieving their repression of FT and promoting flowering [60]. Furthermore, studies have shown a contrasting expression pattern between *miR156* and *miR172*. *MiR172* increases its expression with age, while *miR156* is upstream of *miR172*. By regulating the expression of *SPL9*, *miR156* indirectly controls the expression of *miR172*, thereby regulating plant flowering [59]. This regulatory cascade ensures that flowering occurs only after sufficient vegetative growth, optimizing reproductive success.

### 2.7. Integration of Flowering Pathways

**Figure 1:**
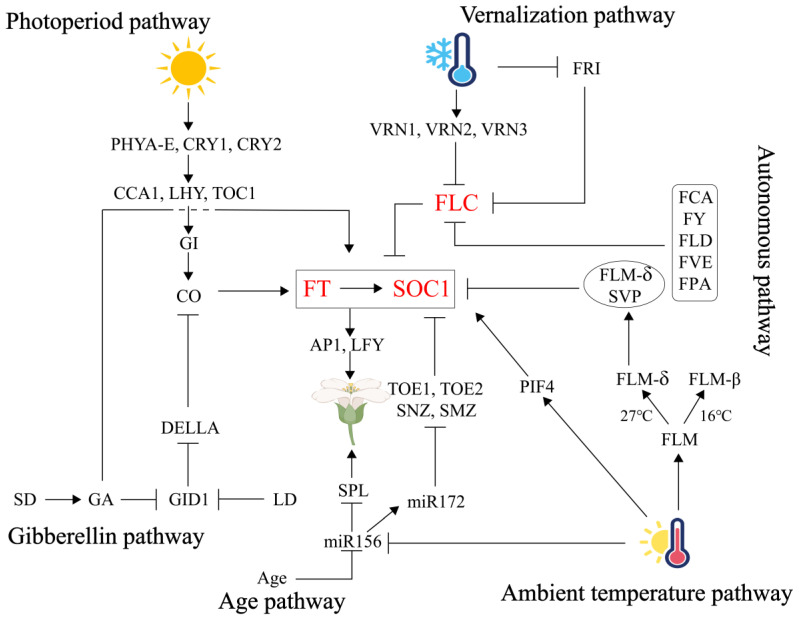
An overview of the six major pathways that regulate floral transition.

Flowering in plants is a genetically regulated, intricate physiological process comprising three stages: floral induction, differentiation of floral primordium, and development of floral organs. Among these, floral induction is the critical phase for plant ontogeny and reproductive success, achieved through the integration of endogenous and exogenous signals via multiple coordinated pathways. As summarized in Figure 1, the photoperiod pathway employs photoreceptor-circadian clock modules (PHY/CRY-CCA1/TOC1) to activate CO, which drives the production of the florigen FT, with its stability regulated by PHYB-mediated phosphorylation. The vernalization pathway relieves repression of FT/SOC1 through low-temperature-induced epigenetic silencing of FLC, synergizing with the autonomous pathway, which utilizes RNA processing (FCA/FY) and chromatin remodeling (FVE/FLD), to establish dual suppression of FLC. The temperature pathway dynamically modulates FT inhibition via temperature-dependent alternative splicing of FLM-β/δ in the SVP-FLM complex, while coordinating with the miR156-SPL module to transduce thermal signals. The gibberellin pathway degrades DELLA proteins (GAI/RGA) under short-day conditions, releasing SPL and PIF4 to activate FT/LFY, while coupling gibberellin signaling with photoperiod regulation through CO interaction. The age pathway relies on a miR156-SPL timer to promote FT expression by suppressing AP2-like transcription factors (TOE/SMZ) via *miR172*, ensuring reproductive transition only after sufficient vegetative growth. These pathways converge at the FT hub, where photoperiod, temperature, gibberellin, and age signals collectively activate FT transcription. The FLC regulatory axis (vernalization/autonomous pathways) and SPL nodes (age/gibberellin/temperature) further form a multi-layered interaction network. Ultimately, the FT-FD protein complex initiates flowering programs in the shoot apical meristem, achieving a dynamic balance between environmental adaptability and developmental robustness (Figure 1).

The photoperiod pathway activates the CO-FT axis via PHY/CRY-CCA1/TOC1 modules; the vernalization/autonomous pathways epigenetically silence FLC to relieve FT/SOC1 repression; the temperature pathway dynamically regulates SVP-FLM-mediated FT inhibition through FLM-β/δ alternative splicing; the gibberellin pathway degrades DELLA proteins to release SPL/PIF4 for FT/LFY activation; and the age pathway suppresses AP2-like transcription factors via the miR156-SPL-miR172 cascade. Signals converge at the FT hub, with the FLC axis and SPL nodes forming a multi-layered network. The FT-FD complex initiates flowering in the shoot apical meristem, balancing environmental adaptability and developmental robustness. The three genes *FT*, *SOC1*, and *FLC* jointly regulate the six major pathways controlling flowering induction, which we have marked in red.

## 3. Epigenetic Control of Flowering Regulation in Plants

Flowering is precisely regulated by a complex interaction of genetic and epigenetic mechanisms. Epigenetic regulation provides a heritable yet flexible framework for environmental adaptation, enabling plants to modulate gene expression in response to seasonal changes. Major epigenetic pathways include histone modifications, DNA methylation, chromatin remodeling, non-coding RNA regulation, and RNA modifications [61]. These mechanisms often converge at key flowering genes such as *FLC*, *FT*, and *SOC1*, orchestrating transcriptional activation or repression through chromatin and RNA-level modifications.

### 3.1. Histone Modifications

Histones form the nucleosome core octamer (H2A, H2B, H3, and H4) that packages 147 bp of DNA [62] with post-translational modifications (PTMs) serving as key epigenetic regulators. Histone alterations are some of the most extensively researched epigenetic pathways in the regulation of flowering. These covalent modifications, including methylation, acetylation, phosphorylation and ubiquitination, among others [4]. Covalent post-translational modifications—such as methylation, acetylation, ubiquitination, and phosphorylation—alter chromatin compaction and accessibility, thereby influencing transcriptional outcomes.

#### Histone Methylation

Histone methylation dynamically regulates chromatin states through balanced “writer-eraser” systems. Histone methylation catalyzed by distinct enzyme families, occurs predominantly on lysine (K) and arginine (R) residues: histone lysine methyltransferases (HKMTases) predominantly contain Su(var)3-9, Enhancer of zeste, Trithorax (SET) or disruptor of telomeric silencing 1-like (DOT1L) domain mediating K-methylation (me1-me3) at conserved positions (e.g., H3K4/9/27/36/79, H4K20), while protein arginine methyltransferases (PRMTases) catalyze R-methylation, generating arginine monomethylation (MMA), asymmetric arginine dimethylation (ADMA), and symmetric arginine dimethylation (SDMA) [62]. In *Arabidopsis thaliana*, 47 SET domain-containing proteins named SET DOMAIN GROUP (SDG) have been classified into functional subgroups: E(z), ASH1, Trithorax, proteins with one SET domain and one PHD domain, Su(var), proteins with a broken SET domain, and proteins without HMTase activity [63]. There are nine PRMTs in total, which are categorized by product specificity as follows: protein arginine methyltransferase 1 (PRMT1), PRMT2, PRMT3, PRMT4, PRMT6, and PRMT8 catalyze MMA and ADMA, whereas PRMT5, PRMT7, and PRMT9 produce MMA and SDMA [64]. Demethylation is mediated by two major enzyme groups: the lysine-specific demethylase (LSD)/JmjC-domain-containing histone demethylases (JHDM), and peptidyl arginine deiminases (PAD4/PADI4)/JmjC domain-containing 6 protein (JMJD6) for arginines, completing the dynamic regulation of chromatin states [65,66].

The epigenetic regulation of plant flowering through histone methylation involves a sophisticated interplay of enzymatic writers, erasers, and readers that dynamically modulate chromatin states at key developmental loci. Central to this process are the antagonistic roles of activating (H3K4me3) and repressive (H3K27me3) methylation marks, which establish bistable chromatin configurations to precisely control flowering time. In *Arabidopsis thaliana*, the Trithorax-group methyltransferase ATX1/SDG27 deposits H3K4me3 at *FLC* and *FT* loci, maintaining transcriptional activation by facilitating RNA polymerase II recruitment and chromatin accessibility [67]. Conversely, Polycomb Repressive Complex 2 (PRC2), containing the E(z) homolog CLF/SDG1, catalyzes H3K27me3 deposition at these loci, enforcing transcriptional silencing through chromatin compaction [68]. This antagonism is further regulated by environmental cues, as demonstrated in vernalization pathways where prolonged cold exposure triggers PRC2-mediated H3K27me3 enrichment at FLC, a process facilitated by lncRNAs (COOLAIR, COLDAIR) and the FCA-SSU72 regulatory module. The dynamic equilibrium between these marks ensures robust silencing of FLC post-vernalization while permitting rapid floral induction under permissive conditions [69].

Emerging evidence highlights the regulatory significance of H3K36me3, deposited by SET DOMAIN GROUP 7 (SDG7), which promotes transcriptional elongation and chromatin remodeling through interactions with the SWR1 complex subunit SWC6 [70]. This modification facilitates H2A.Z variant incorporation at FLC chromatin, sustaining transcriptional activity and delaying flowering. Temperature-dependent regulation is exemplified in Brassica rapa, where the H3K36me2/3 demethylase BrJMJ18 dissociates from BrFLC3 chromatin under elevated temperatures, lifting repression and delaying flowering. Similarly, thermosensitive H3K27me3 demethylases such as JMJ13 integrate photoperiodic and thermal signals. The *jmj13* mutants exhibits early flowering under long-day conditions and its consistently attenuated expression under short-day or high-temperature conditions leads to the derepression of FT, thereby initiating flowering [71].

Cross-species conservation of these mechanisms is evident in winter wheat (Triticum aestivum), where vernalization-induced H3K27me3 depletion at VRN1 releases transcriptional repression, while H3K36me3 maintains transcriptional memory post-cold exposure [72]. In kiwifruit (*Actinidia chinensis*), low-temperature induction of H3K4me3 at AcFLC homologs underscores the evolutionary conservation of methylation-mediated vernalization responses [73]. The regulatory network extends to FT through PRC2-mediated H3K27me3 silencing and redundant H3K4me3 demethylases (AtJMJ4/ELF6), which collectively fine-tune floral integrator expression [74,75].

Despite these advances, critical questions remain regarding the spatiotemporal coordination of methylation marks, the identity of epigenetic sensors translating environmental signals into chromatin modifications, and the mathematical modeling of cross-regulatory networks. Future studies integrating multi-omics approaches and single-cell epigenomics will be essential to unravel the complexity of histone methylation in flowering time regulation, offering novel strategies for crop improvement in changing climates.

### 3.2. Histone Acetylation

Histone acetylation, a reversible post-translational modification facilitated by histone acetyltransferases (HATs), is crucial for regulating chromatin accessibility and transcriptional activity during floral transition. This dynamic process is counterbalanced by histone deacetylases (HDACs), which restore chromatin compaction through acetyl group removal, establishing an acetylation-deacetylation equilibrium critical for flowering time regulation. The HAT superfamily is classified into four conserved subfamilies: GCN5-related N-acetyltransferase (GNATs), MOZ-YBF2/SAS3-SAS2-TIP60 (MYST), CREB-binding (p300/CBP), and TBP-associated factor 1 (TAF1) [76], while HDACs encompass Reduced Potassium Dependence 3 (RPD3)/Histone Deacetylase 1 (HDA1), plant-specific Histone Deacetylase 2 (HD2), and NAD^+^-dependent Silent Information Regulator 2 (SIR2) families [77]. GNAT catalyzes H3K14 or H3K12 sites; MYST catalyzes H4K5 sites; CBP/p300 acts on all sites that can undergo acetylation; and TAF1 involves promoter-proximal regulation. In Arabidopsis, the vernalization pathway exemplifies this regulatory interplay, where VIN3, the HATs, acetylates H3K27 at the FLC locus during early cold exposure, enhancing transcriptional activation [78]. Prolonged cold, however, recruits HDAC complexes containing FLD and FVE to deacetylate FLC chromatin, facilitating Polycomb-mediated repression via H3K27me3 deposition and ensuring floral competence. Disruption of FLD or FVE results in hyperacetylated FLC chromatin, elevated FLC expression, and delayed flowering, underscoring the necessity of HDAC activity in vernalization memory [79].

Beyond FLC regulation, histone acetylation intersects with multiple flowering pathways. The RPD3/HDA1-family deacetylase HDA5 suppresses FLC expression by maintaining histone H3 hypoacetylation, with hda5 mutants exhibiting concurrent increases in H3 acetylation and H3K4me3, revealing cross-talk between acetylation and methylation systems [80]. Photoperiodic control involves HDA9, which represses FT and AGL19 under non-inductive short-day (SD) conditions; hda9 loss derepresses these loci, accelerating flowering [81]. Metabolic-epigenetic integration is demonstrated by ALDEHYDE DEHYDROGENASE 3F1 (ALDH3F1), which enhances H3K9 acetylation at FLC to delay flowering, linking cellular redox states to chromatin modification [82]. Conservation of these mechanisms is evident in rice, where the OsMRG702-OsMRGBP complex regulates heading date through H4K5 acetylation dynamics, with knockout lines showing reduced H4 acetylation and delayed flowering [83].

These findings collectively underscore the dual function of acetylation as both an activator and a tunable repressor in flowering regulation, contingent on spatiotemporal context and environmental inputs. Unresolved questions persist regarding the specificity of HAT/HDAC isoforms, the molecular sensors translating temperature fluctuations into acetylation dynamics, and the mechanistic integration of metabolic signals with chromatin states. Addressing these gaps through advanced epigenomic profiling and cross-species comparative studies will deepen our understanding of histone acetylation networks, offering novel strategies for manipulating flowering time in agronomically important species under climate change scenarios.

#### Histone Ubiquitination and Phosphorylation

Histone ubiquitination, a dynamic and reversible post-translational modification, regulates chromatin states through coordinated actions of ubiquitinating and deubiquitinating enzymes. The ubiquitination cascade involves three sequential enzymatic steps: ubiquitin activation by E1, conjugation by E2, and substrate-specific ligation by E3, exemplified in Arabidopsis by the HUB1/HUB2 E3 ligase complex that mediates monoubiquitination of histone H2B at lysine 143 (H2Bub1) [84]. This modification facilitates transcriptional activation of *FT*, a key floral integrator, thereby promoting flowering. Similarly, in *Arabidopsis*, the loss of E3 ubiquitin ligase homologs HISTONE MONOUBIQUITINATION1 (HUB1) and HUB2 reduces H2B monoubiquitination (H2Bub1) levels, consequently decreasing FLC expression and leading to early flowering [85]. Conversely, deubiquitination enzymes (DUBs)—classified into cysteine proteases (UBP, UCH, OTU, MJD) and metalloproteases (JAMM)—antagonize this process [86]. For instance, UBP26 removes H2Bub1 at FLC chromatin, reducing its expression and accelerating flowering, highlighting the delicate balance between ubiquitination and deubiquitination in flowering time control [87].

Beyond ubiquitination, other PTMs such as phosphorylation also critically regulate flowering. Kinases and phosphatases dynamically modify histones to modulate chromatin states. The *Arabidopsis* MUT9P-LIKE KINASE 4 (MLK4) phosphorylates histone H3 at threonine 3 (H3T3ph), inducing chromatin compaction and FLC silencing to accelerate flowering [88]. Similarly, SR-specific kinase II (SRPKIIs) regulates FLC expression and alternative splicing by phosphorylating serine/arginine-rich (SR) proteins, linking RNA processing to epigenetic control [89].

These modifications intersect with broader flowering pathways, including vernalization, photoperiod, and autonomous regulation, primarily through FT and FLC modulation. The vernalization pathway, for instance, integrates H2Bub1 dynamics with Polycomb-mediated H3K27me3 deposition at FLC, while phosphorylation-mediated chromatin restructuring fine-tunes transcriptional outputs. Emerging evidence underscores cross-talk between ubiquitination, phosphorylation, and other histone modifications, as seen in the coordinated regulation of H2A.Z incorporation and H3K36me3 deposition. However, critical gaps remain in understanding the spatiotemporal coordination of these modifications, the specificity of E3 ligase-DUB interactions, and the mechanistic basis of phosphorylation-dependent chromatin remodeling. Future studies employing multi-omics approaches and structural analyses will be essential to unravel these networks, offering novel insights into epigenetic engineering of flowering time for agricultural resilience.

### 3.3. DNA Methylation

DNA methylation, a conserved epigenetic modification, regulates gene expression and chromatin states through the establishment of 5-methylcytosine (5-mC) in three sequence contexts: symmetric CG and CHG, and asymmetric CHH (H = A, T, or C) [90]. This process is catalyzed by DNA methyltransferases (DNMTs) utilizing S-adenosylmethionine (SAM) as a methyl donor, while active demethylation by glycosylases such as REPRESSOR OF SILENCING 1 (ROS1) and DEMETER (DME) removes methyl groups to enable transcriptional plasticity. In plants, methylation occurs via two primary modes: maintenance methylation (preserving CG and CHG patterns through MET1 and CMT3, respectively) and de novo methylation (establishing CHH methylation via the RNA-directed DNA methylation (RdDM) pathway, involving small RNAs and DOMAINS REARRANGED METHYLTRANSFERASE 2 (DRM2)) [91]. These dynamics are critical for repressing transposons, stabilizing genomes, and regulating developmental transitions, including floral induction.

In the vernalization pathway, the *drdd* quadruple mutant of *Arabidopsis* demethylases (DME, DML2, DML3, and ROS1) causes hypermethylation at the 5′ region of FLC due to demethylase deficiency, which blocks transcription factor binding, disrupts transcription, and ultimately leads to premature flowering [92].

In the autonomous pathway, CG methylation maintained by MET1 ensures stable silencing of FLC, a central floral repressor. MET1 loss triggers FLC reactivation and delayed flowering, highlighting CG methylation’s role in transcriptional memory [93]. Conversely, vernalization-induced flowering involves CHH methylation dynamics, where cold exposure elevates CHH methylation at the VRN1 promoter, correlating with its activation. This pathway further integrates AGAMOUS-LIKE 20 (AGL20), whose expression positively associates with CHH methylation levels during vernalization progression [94]. Photoperiodic regulation employs similar mechanisms, as demonstrated in chrysanthemum (*Chrysanthemum morifolium*), where 5-azacytidine (a DNMT inhibitor) or MET1 silencing reduces global methylation, altering flowering timing—a finding replicated across species, including Dactylis glomerata, where exogenous methyl donors accelerate flowering via hypermethylation [95].

The age pathway in Moso bamboo (*Phyllostachys edulis*) illustrates developmental-stage-specific methylation switching: hypermethylation at CHH sites silences SOC1 during vegetative growth, while reproductive transition involves CHH hypomethylation, enabling PeSPL3f-mediated SOC1 activation [96]. Gibberellin (GA) signaling intersects with methylation networks, as GA treatment reduces global cytosine methylation in rhododendron buds, promoting early flowering—a phenomenon mirrored in tree peony (Paeonia suffruticosa), where developmental demethylation activates PsFT expression. These findings collectively suggest that DNA hypomethylation generally serves as a transcriptional activation signal, with demethylation events at key loci (e.g., *FT*, *SOC1*) integrating environmental, hormonal, and age-related cues to trigger flowering [97].

Despite these advances, critical gaps persist in understanding sequence-specific recruitment of methylation/demethylation machinery, the mechanistic integration of hormonal signals (e.g., GA) with epigenetic modifiers, and the conservation of these networks between annual and perennial species. Furthermore, while low methylation correlates with floral activation, the precise regulatory logic governing context-dependent effects, such as CHH methylation’s dual roles in repressing SOC1 yet activating VRN1, remains unresolved. Future studies leveraging single-cell methylomics and cross-species comparative approaches will be essential to decode the spatiotemporal precision of DNA methylation in flowering regulation, offering novel strategies for manipulating reproductive timing in crop species under climate change scenarios.

### 3.4. Chromatin Remodeling

Chromatin remodeling complexes, ATP-dependent molecular machines that modulate nucleosome positioning and chromatin accessibility, play pivotal roles in flowering time regulation through dynamic reorganization of chromatin architecture. These complexes are classified into four major families according to their catalytic ATPase subunits: SWItch/Sucrose Non-Fermentable (SWI/SNF), inositol requiring mutant 80/Swi2/Snf2-related gene 1 (INO80/SWR1), Imitation Switch (ISWI), and Chromodomain-Helicase-DNA-binding (CHD), each exerting distinct yet interconnected effects on floral integrator genes [98].

The SWI/SNF family, exemplified by the *Arabidopsis* BRAHMA (BRM) and SPLAYED (SYD) ATPases, regulates flowering by repressing key floral repressors [99]. BRM directly binds the promoter of SHORT VEGETATIVE PHASE (SVP), suppressing its expression to derepress CONSTANS (CO) and FLOWERING LOCUS T (FT), thereby accelerating flowering [100]. Concurrently, SYD interacts with LEAFY to remodel chromatin at *AGAMOUS* (*AG*) loci, regulating floral organ identity [101]. These ATPases also collaborate redundantly to activate APETALA3 (AP3) and AG expression, demonstrating functional synergy in floral development [102]. The SWI/SNF complex further incorporates BRG1/BRM-associated factor 60 (BAF60) homologs, which facilitate chromatin loop formation at FLC to modulate its expression, while BRM recruits GATA, Nitrate-inducible, Carbon-metabolism-involved (GNC) transcription factors to repress SUPPRESSOR OF OVEREXPRESSION OF CONSTANS 1 (SOC1), integrating environmental and developmental signals [103].

The INO80/SWR1 family orchestrates histone variant exchange, replacing canonical H2A with H2A.Z to fine-tune transcriptional outputs [104]. In *Arabidopsis*, the SWR1 complex—comprising PHOTOPERIOD-INDEPENDENT EARLY FLOWERING 1 (PIE1), ACTIN-RELATED PROTEIN 6 (ARP6), and SWR1 COMPLEX SUBUNIT 6 (SWC6)—deposits H2A.Z at FLC chromatin, stabilizing repressive states during vernalization. *pie1* mutants exhibit FLC downregulation and early flowering, highlighting SWR1′s role in maintaining floral repression [105]. Conversely, EARLY BOLTING IN SHORT DAYS (EBS) partners with SWR1 to inhibit FT expression through chromatin compaction, delaying flowering under non-inductive conditions [106]. ISWI-family remodelers, including Chromatin Remodeling 11 (CHR11) and Chromatin Remodeling 17 (CHR17), regulate chromatin accessibility by interacting with DDT domain-containing proteins RINGLET (RLT), maintaining nucleosome spacing uniformity at flowering loci such as *FT*, *SOC1*, and *FLC*; loss of ISWI function disrupts nucleosome organization at *SEPALLATA* (*SEP*) and *FRUITFUL* (*FUL*) loci, impairing floral organ development [107]. In contrast, the ISWI complex subunit ARID5 employs dual ARID-PHD domains to recognize H3K4me3 marks and specific DNA sequences, ensuring targeted remodeling, and by directly targeting genes including *FLC*, *SVP*, *FT*, and *SEP3*, it modulates the expression of key regulators to control the floral transition [108].

CHD-family remodelers, represented by PICKLE (PKL), antagonize Polycomb-mediated repression at FT by reconfiguring chromatin architecture. PKL also enhances CO’s transcriptional activation of FT through direct interaction, illustrating cross-talk between photoperiodic signaling and chromatin states [109]. This functional diversification underscores CHD’s role in balancing transcriptional activation and repression [110].

These complexes collectively regulate flowering through three mechanistic tiers: (1) direct chromatin restructuring at floral integrators (FLC, FT, SOC1), (2) integration of environmental cues (vernalization, photoperiod) via histone variant exchange and nucleosome repositioning, and (3) coordination with other epigenetic systems (Polycomb, histone acetylation). However, unresolved questions persist regarding tissue-specific complex composition, ATPase recruitment specificity, and evolutionary conservation of remodeling mechanisms across monocots and dicots. Elucidating these dynamics through structural biology and genome-wide chromatin mapping will advance our ability to engineer flowering time in crops, addressing challenges posed by climate variability.

### 3.5. Non-Coding RNAs (ncRNAs)

ncRNAs, a varied category of regulatory molecules, orchestrate flowering time through intricate interactions with chromatin modifiers, transcription factors, and RNA processing machinery. These ncRNAs are broadly categorized into housekeeping RNAs (e.g., tRNA, rRNA) and regulatory RNAs, with the latter encompassing long non-coding RNAs (lncRNAs), microRNAs (miRNAs), and small interfering RNAs (siRNAs) [111]. Among these, lncRNAs and miRNAs have emerged as central players in flowering regulation, integrating environmental and developmental cues to fine-tune floral transitions.

Long non-coding RNAs (lncRNAs), constituting 80–90% of ncRNAs, modulate gene expression via chromatin remodeling and transcriptional interference [112]. At the FLOWERING LOCUS C (FLC) locus, antisense lncRNAs COOLAIR and COLDAIR recruit Polycomb Repressive Complex 2 (PRC2) to deposit repressive H3K27me3 marks, silencing FLC during vernalization [113]. COOLAIR interacts with the RNA-binding protein FCA to stabilize PRC2 binding, while COLDAIR, transcribed from FLC introns, directly scaffolds PRC2 to the locus. Cold-induced transcription factor WRKY63 enhances their expression, linking temperature sensing to epigenetic silencing [114]. MicroRNAs (miRNAs), 20–24 nucleotide non-coding RNAs processed by DICER-LIKE1 (DCL1), HYPONASTIC LEAVES1 (HYL1), and SERRATE (SE), post-transcriptionally repress target mRNAs via sequence complementarity [115,116]. The miR156/miR172 module functions as a conserved age-dependent flowering switch: high *miR156* levels in juvenile plants suppress SQUAMOSA PROMOTER BINDING PROTEIN-LIKE (SPL) transcription factors, delaying flowering by inhibiting FT expression [117]. As plants mature, declining *miR156* permits SPL accumulation, which activates *miR172* to repress APETALA2 (AP2)-like floral inhibitors, forming a miR156/157-SPLs-miR172-AP2 cascade that promotes reproductive transition. This module also integrates temperature signals, with low temperatures modulating SPL and AP2 expression to adjust flowering timing [118].

These ncRNA pathways intersect with other epigenetic systems. For instance, lncRNA-mediated PRC2 recruitment synergizes with histone methylation to stabilize FLC silencing, while miR156/172 dynamics coordinate with chromatin remodeling complexes to regulate FT accessibility. Furthermore, temperature-responsive lncRNAs and miRNAs bridge environmental sensing to chromatin state changes, exemplifying the integration of external cues with developmental programs. Despite these advances, key questions persist regarding the tissue-specificity of ncRNA action, the conservation of regulatory networks across monocots and dicots, and the mechanistic basis of lncRNA-chromatin interactions. Resolving these through spatial transcriptomics and cross-species comparative studies will deepen our understanding of ncRNA-mediated flowering regulation, offering novel strategies for crop improvement in variable climates.

### 3.6. RNA Modifications

RNA modifications, particularly N6-methyladenosine (m^6^A), have emerged as dynamic regulators of flowering time by influencing mRNA stability, splicing, and translational efficiency. m^6^A, the most prevalent internal RNA modification in eukaryotes, is deposited by a conserved methyltransferase complex (MTA/MTB/FIP37/VIRILIZER/HAKAI) using S-adenosylmethionine (SAM) as a methyl donor and removed by demethylases (ALKBH9B/ALKBH10B) [119,120]. These marks are recognized by YTH domain reader proteins (ECT2, ECT3, ECT4, and CPSF30-L), which direct mRNA fate through selective binding [121,122,123]. In *Arabidopsis thaliana*, m^6^A exhibits distinct distribution patterns, concentrated near start/stop codons and 3′ untranslated regions (UTRs), with conserved RRACH motifs diverging from mammalian GRACHA motifs, reflecting evolutionary specialization in plant RNA metabolism [124].

The flowering regulator FLC exemplifies the dual function of m^6^A in transcript stability. The methyltransferase FIONA1 mediates m^6^A modification on *FLC* mRNA, stabilizing its transcripts to delay flowering, while ALKBH10B demethylates *FT* mRNA, enhancing its stability to accelerate floral transition. FIONA1 further integrates photoperiodic signals by modulating circadian clock genes (*CCA1*, *LHY*, *TOC1*, *LUX*) and key floral integrators (CO, SPL3, SEP3), illustrating its pleiotropic role in flowering regulation [125]. Conversely, ALKBH10B deficiency increases m^6^A levels on *SPL3/9* and *FT* transcripts, promoting their degradation and delaying flowering [126]. Reader proteins amplify these effects: *ect2/3/4* triple mutants exhibit delayed bolting and floral defects due to impaired SOC1 mRNA processing, while CPSF30-L loss extends SOC1 3′ UTRs, accelerating transcript decay and delaying flowering [126]. Another m^6^A reader, FLK, promotes flowering by recognizing m^6^A modifications on *FLC* mRNA. This recognition leads to the suppression of FLC expression through mechanisms involving mRNA stability and splicing [40]. Recent discoveries highlight that m^6^A functions in coordination with chromatin-based regulation. For instance, the NERD complex links m^6^A-modified RNAs to transcriptional gene silencing, providing a mechanistic bridge between RNA modification and histone methylation. This interplay suggests that m^6^A not only tunes *FLC* transcript stability but also interfaces with nuclear chromatin states, establishing multilayered regulatory feedback loops [127].

Furthermore, cross-species studies reveal both conservation and divergence in m^6^A-mediated regulation. In rice, m^6^A modification is recognized by OsYTH10, which stabilizes the transcripts of key flowering genes *OsGI* and *OsDTH7*, thereby regulating flowering time under long-day conditions [128]. In cotton, m^6^A modification—mediated through demethylation by GhALKBH5—modulates the mRNA stability of key photoperiod pathway genes *GhADO3* and *GhAGL24*. This subsequently affects the expression of the floral integrator GhFT1, ultimately determining cotton’s flowering response to long-day conditions [129]. Collectively, m^6^A acts as a flexible molecular switch that integrates epigenetic, hormonal, and environmental cues to modulate floral timing, representing a promising target for epigenetic breeding strategies.

Beyond m^6^A, emerging evidence implicates 5-methylcytosine (m^5^C) and pseudouridine (Ψ) in flowering regulation, though their mechanisms remain elusive [130]. Technical challenges in RNA modification detection have historically hindered progress, but recent advances reveal m^6^A’s centrality in photoperiodic pathways. However, its roles in vernalization, autonomous, and gibberellin-mediated flowering pathways are underexplored, highlighting critical knowledge gaps. Evolutionary divergence in m^6^A motifs and reader specificity between plants and animals further underscores the need for species-specific mechanistic studies.

Collectively, RNA modifications represent a versatile regulatory layer integrating transcriptional and post-transcriptional control of flowering. Future research leveraging single-molecule sequencing and cross-species epigenomics will unravel the spatiotemporal dynamics of these modifications, their crosstalk with chromatin states, and their potential as targets for precision breeding in crops. Addressing these questions will deepen our understanding of how RNA epitranscriptomics fine-tunes reproductive transitions in response to environmental and developmental signals.

### 3.7. Integration of Epigenetic Mechanisms

**Figure 2:**
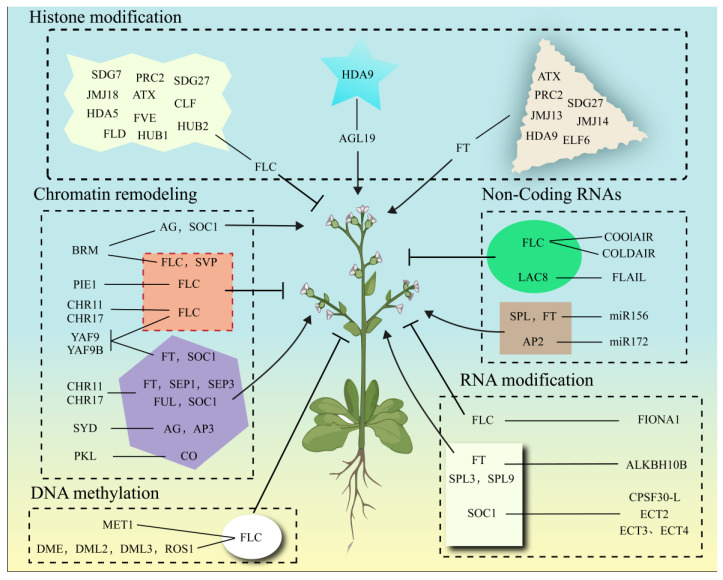
The epigenetic regulation of floral transition.

The epigenetic regulation of flowering represents a sophisticated, interconnected network where histone modifications, DNA methylation, chromatin remodeling, RNA modifications, and non-coding RNAs synergistically fine-tune gene expression (Table 1; Figure 2). At loci such as FLC and FT, combinatorial epigenetic marks—including H3K27me3 deposition by PRC2 and MET1-mediated CG methylation—establish bistable chromatin states that reinforce transcriptional repression or activation. Chromatin remodelers (e.g., SWI/SNF, INO80/SWR1) further coordinate with histone modifiers to dynamically reposition nucleosomes, thereby facilitating the recruitment of transcriptional machinery or silencing complexes. Concurrently, RNA modifications like m^6^A and lncRNAs add regulatory layers by modulating mRNA stability (e.g., FIONA1 stabilizing *FLC* transcripts) or recruiting epigenetic effectors (e.g., COOLAIR guiding PRC2 to FLC). This multi-tiered regulation ensures robust integration of environmental cues (photoperiod, temperature) and endogenous signals (hormonal, developmental) to optimize flowering timing.

Plant flowering is coordinately regulated by epigenetic modifications: histone modifications (methylation, acetylation, ubiquitination) dynamically balance chromatin states through “writer-eraser” systems, integrating photoperiod and vernalization signals; DNA methylation (CG/CHG/CHH) maintains gene silencing or activation, mediating environmental memory and developmental transitions; chromatin remodeling complexes (SWI/SNF, INO80, etc.) reorganize nucleosome positioning to regulate accessibility of key genes (*FLC*/*FT*/*SOC1*); non-coding RNAs (lncRNAs/miRNAs) recruit epigenetic modifiers or target mRNAs for temporal control; and RNA modifications (m^6^A) fine-tune transcript stability and translational efficiency. Solid lines with arrowheads indicate promotion, while solid lines with flat ends denote inhibition.

Despite advances, critical gaps persist in understanding the spatiotemporal coordination of these mechanisms. Key questions include how cross-talk between DNA methylation, histone modifications, and RNA epitranscriptomics establishes epigenetic memory, and how chromatin remodelers precisely target specific loci. Emerging tools such as CRISPR-d Cas9-based epigenetic editors and single-cell multi-omics promise to unravel these complexities. Furthermore, the agricultural potential of epigenetic engineering remains underexploited. For instance, modulating m^6^A via demethylases like ALKBH10B could enhance FT expression to accelerate flowering in crops, while manipulating SWR1-mediated H2A.Z deposition might improve vernalization responses in temperate cereals. However, technical challenges—particularly in RNA modification detection and tissue-specific epigenetic editing—must be addressed to realize these applications.

Climate change underscores the urgency of this research. Epigenetic plasticity enables plants to adjust flowering time under environmental stress, yet the mechanisms underlying this adaptability are poorly understood. Studies in tomato reveal that the m^6^A demethylase SlALKBH9B suppresses drought-induced flower abscission through the ethylene biosynthesis pathway, thereby shedding light on the epigenetic mechanisms governing organ abscission during stress responses [131]. Studies in rice demonstrate that m^6^A modification regulates drought tolerance and yield, suggesting epigenetic traits could be harnessed for climate-resilient crops [132]. Similarly, Populus trichocarpa engineered with altered DNA methylation exhibits enhanced drought resistance, highlighting the translational potential of epigenetic interventions [133]. However, most research remains confined to model systems; expanding to crops like maize and wheat is essential.

This integrative view underscores that epigenetic modifications serve as dynamic layers of transcriptional memory, balancing environmental flexibility with developmental stability. Understanding this interplay provides novel avenues for manipulating flowering time through targeted epigenetic editing.

### 3.8. Future Perspectives

Recent advances in multi-omics technologies—including ATAC-seq, ChIP-seq, and m^6^A-seq—have revealed intricate cross-regulatory networks connecting chromatin states and RNA metabolism. Future research should aim to (1) integrate single-cell epigenomics to dissect tissue-specific flowering dynamics; (2) employ CRISPR/dCas9-based epigenetic editors for precise modification of histone marks and DNA methylation at flowering loci; (3) explore the interplay between m^6^A and chromatin modifications to uncover higher-order regulatory mechanisms; and (4) translate these discoveries into crop breeding programs, optimizing flowering time and stress resilience. By bridging fundamental insights with translational applications, epigenetic research will continue to redefine our understanding of plant developmental plasticity and adaptation.

Epigenetic regulation forms the molecular backbone of floral transition, integrating genetic, environmental, and developmental cues. By coordinating multiple layers—from chromatin remodeling to RNA modification—plants achieve remarkable flexibility in reproductive timing. A comprehensive understanding of these processes will not only deepen our insight into plant developmental biology but also enable precision control of flowering through targeted epigenetic breeding, paving the way for sustainable crop adaptation in a changing climate.

## Figures and Tables

**Table 1:** Summary of epigenetic factors in flowering pathway in plants.

Genes	Functions	Modification Type	Regulated Gene
*ATX/SDG27*	Histone methyltransferases	Histone modifications	*FLC*, *FT*
*SDG7*	Histone methyltransferases	Histone modifications	*FLC*
*JMJ18*	Histone demethyltransferases	Histone modifications	*FLC*
*JMJ13*	Histone demethyltransferases	Histone modifications	*FT*
*PRC2*	Histone methyltransferases	Histone modifications	*FLC*, *FT*
*CLF*	Histone methyltransferases	Histone modifications	*FLC*
*JMJ14* *, ELF6*	Histone demethyltransferases	Histone modifications	*FT*
*FLD*, *FVE*	Histone acetyltransferases	Histone modifications	*FLC*
*VIN3*	Histone acetyltransferases	Histone modifications	*FLC*
*HDA5*	Histone deacetylases	Histone modifications	*FLC*
*HDA9*	Histone deacetylases	Histone modifications	*FT*, *AGL19*
*HUB1*, *HUB2*	ubiquitin-ligase	Histone modifications	*FLC*
*MET1*	DNA methyltransferase	DNA methylation modification	*FLC*
*DME*, *DML2*, *DML3*, *ROS1*	DNA demethylase	DNA methylation modification	*FLC*
*BRM*	SWI2/SNF2 Chromatin remodeling complex subunits	Chromatin remodeling	*FLC*, *SVP*, *SOC1*, *AG*
*SYD*	SWI/SNF Chromatin remodeling complex subunits	Chromatin remodeling	*AG*, *AP3*
*CHR11*, *CHR17*	ISWI chromatin remodeling proteins	Chromatin remodeling	*FT*, *SEP1*, *SEP3, FUL*, *SOC1*, *FLC*
*PIE1*	SWR1 complex subunit	Chromatin remodeling	*FLC*
*YAF9A*, *YAF9B*	INO80/SWR1 chromatin remodeling complex subunits	Chromatin remodeling	*FLC*, *FT*, *SOC1*
*PKL*	CHD3 subfamily chromatin remodeling factors	Chromatin remodeling	*CO*
*COOLAIR*	lncRNA	Non-coding RNA	*FLC*
*COLDAIR*	lncRNA	Non-coding RNA	*FLC*
*FLAIL*	lncRNA	Non-coding RNA	*LAC8*
*miR156*	miRNA	Non-coding RNA	*SPL*, *FT*
*miR172*	miRNA	Non-coding RNA	*AP2*
*FIONA1*	m^6^A methyltransferases	m^6^A modifications	*FLC*
*ALKBH10B*	m^6^A demethyltransferases	m^6^A modifications	*FT*, *SPL3*, *SPL9*
*ECT2/3/4*	m^6^A reader	m^6^A modifications	*SOC1*
*CPSF30-L*	m^6^A reader	m^6^A modifications	*SOC1*

## Data Availability

No new data were created or analyzed in this study.
